# Adolescent Internet Abuse: A Study on the Role of Attachment to Parents and Peers in a Large Community Sample

**DOI:** 10.1155/2018/5769250

**Published:** 2018-03-08

**Authors:** Giulia Ballarotto, Barbara Volpi, Eleonora Marzilli, Renata Tambelli

**Affiliations:** Department of Dynamic and Clinical Psychology, University of Rome “La Sapienza”, Via degli Apuli 1, Rome, Italy

## Abstract

Adolescents are the main users of new technologies and their main purpose of use is social interaction. Although new technologies are useful to teenagers, in addressing their developmental tasks, recent studies have shown that they may be an obstacle in their growth. Research shows that teenagers with Internet addiction experience lower quality in their relationships with parents and more individual difficulties. However, limited research is available on the role played by adolescents' attachment to parents and peers, considering their psychological profiles. We evaluated in a large community sample of adolescents (*N* = 1105) the Internet use/abuse, the adolescents' attachment to parents and peers, and their psychological profiles. Hierarchical regression analyses were conducted to verify the influence of parental and peer attachment on Internet use/abuse, considering the moderating effect of adolescents' psychopathological risk. Results showed that adolescents' attachment to parents had a significant effect on Internet use. Adolescents' psychopathological risk had a moderating effect on the relationship between attachment to mothers and Internet use. Our study shows that further research is needed, taking into account both individual and family variables.

## 1. Introduction

During the last decade, there has been an enormous development and diffusion of new forms of Internet-information and communication technology, such as social media, personal computer, mobile or cellular phone, and other devices [1]. Adolescents and young adults represent the most users of these different tools [2, 3], and the main purpose of use is social interaction and interpersonal communication [4]. However, research has underlined that some adolescents tend to use Internet excessively or in a maladaptive way, especially to manage psychological suffering [5] and negative emotions associated with problematic relationships with parents and peers [6].

During adolescence numerous changes occur, abilities functional to self-regulation are still relatively immature [7]: recent studies on adolescents' brain development highlighted that emotion-activating experiences (including the over- or misuse of Internet) could interfere with significant modifications of brain regions and systems, such as the prefrontal cortex and the limbic system. These areas play a key role in the regulation of emotions and in the evaluation of the risk [8, 9] and could be responsible for adolescents' general tendency to risk taking and impulsivity [10, 11]. Therefore, it may explain also adolescents' vulnerability to excessive use of Internet, especially with the lack of self-regulatory strategies [12] and when parents are unable to offer an external regulation to their offspring. Moreover, from a developmental point of view, young people go through many developmental tasks and, despite the time spent on the Internet, this could have a different function for them [13]. Specifically, in the early adolescence (from 12 to 14 years; [14]), numerous physical and emotional changes triggered by puberty occur and there is a progressive increase of reflection on emotional experiences [15]; middle adolescence (from 15 to 17 years) is characterized by the onset of adolescent's psychological separation from parents and of the concomitant research of new significant extrafamilial figures, first of all peers; finally, during the late adolescence (from 18 to 20 years), youth have to define their personal, social, and sexual identities [14, 16, 17]. Thus, Internet gives youth also the opportunity to experiment and explore important adolescent-phase questions, including identity, autonomy, and sexuality [13, 18].

Beyond the numerous advantages offered by the new forms of technology, recent research has evidenced that adolescent population had a higher risk to develop Internet problematic behavior, a condition defined as Internet addiction (IA) [19]. However, scientific literature in this field is contrasting [20, 21]: the term IA [19] has also been questioned and other authors have used definitions such as compulsive use of the Internet, problematic use of the web, and pathological use of the Internet [22, 23]. IA has been conceptualized as an impulse-control disorder [24], often described as a behavioral addiction [25], which is characterized by an excessive or uncontrolled Internet use that leads to functional impairment and feelings of marked distress [26]. Despite the growing interest shown in considering IA as a specific diagnosis of behavior addiction, to date Section III of DSM-5 [27] included only an Internet-related condition (i.e., Internet gaming disorder [IGD]). However, given that research evidences on IA are yet inconsistent, editors recommended future researches in this field [27].

Among adolescent and young adult population, epidemiological research reported a prevalence ranging from 1% to 18% [25, 28, 29], with higher rates among males [30]. Interestingly, a broad percentage of adolescents (approximately from 12% to 20%) reported an excessive use of Internet, unsatisfying all criteria for IA [31, 32].

However, although the literature has been increasingly interested in the possible impact of Internet use/abuse on adolescent's development, limited research is available on the role played by adolescents' quality of attachment to parents and peers, considering their psychological profiles.

In accordance with the Developmental Psychopathology [33], clinical and subclinical psychological difficulties in adolescence are postulated to be the result of dynamic interaction of different type of protective and/or risk factors, with a central role played by both individual vulnerability and the quality of relationships with parents and friends. Moreover, difficulties in relationships with parents and friends may have bidirectional relationships with psychological difficulties.

During adolescence, there is an increased need for autonomy and independence from parents. Consequently, in these different phases adolescents have to renegotiate their relationships with them [34, 35] and the acceptance from peers became more influential than in earlier ages. However, the quality of relationship with parents remains a fundamental aspect for adolescents' development and adjustment [36–38] and for a good result of transition to autonomy and adulthood [39].

Attachment theory [40] offers a valid model to understand the protective or risky role played by relationship with parents in the development of adolescents' addictive online behavior. In accordance with Armsden and Greenberg [41] adolescents' perceptions of attachment to their parents reflect their perceived emotional closeness and support from attachment figures, in terms of involvement, trust in the accessibility and responsiveness of them, warmth, and nurturance [41]. In particular, in this perspective a problematic Internet use may be considered as a maladaptive strategy used by adolescent to cope with negative emotions associated with situations in which attachment system is activated and to reduce the distress this produces in them [42]. Thus, they may spend excessive time on Internet to avoid the emotional distress that results from attachment situations or engage in online rewarding activities to replace adverse relationship experiences [43, 44].

In particular, it has been evidenced that low attachment to parents is associated with higher time spent on Internet [4, 45] and risky online activities [46]. Furthermore, higher Internet use is associated with less family time [47], poorer quality of family relationship [48], lower maternal relationships [49], and higher paternal alienation [50], while IA was associated with other adverse conditions like parent-adolescent conflict [51], marital conflict [46], and low satisfaction with family functioning [52, 53].

Adolescents that feel that their relationships with parents are cold, not supportive, and emotionally unavailable may excessively use Internet to search for alternative social support, especially by peers [2, 54]. Indeed, given that relationships with peer become higher priority during adolescence, also the quality of attachment to friends may exert a great influence on adolescent's Internet use [50, 55]. However, to date research has shown mixed findings in the association between IA and the quality of relationships with friends. Research has reported that a low quality of peer attachment was associated with high Internet use [56] and IA [46, 57], but other studies have also found that a good quality of relationship with friends was associated with high Internet use [58] and IA [43], especially for entertainment and social-interaction issues [59]. Overall these findings suggest that problematic relationships with peers, characterized by feelings of isolation, anger, and detachment, could lead to adolescents spending excessive time in Internet to seek refuge in a virtual world in which they could establish “fake” social interactions and friendship [57]. At the same time, also attachment security to peers, in which adolescents experience trust and a good quality of involvement and verbal communication with them, could represent a risk factor for IA, for example, by being involved in activities in which their peer group is engaged [43, 46].

As regards the role played by psychological functioning, several studies, rooted in genetic and epigenetic research, have underlined that adolescents that excessively use Internet often had a wide range of psychosocial correlations, such as impulsivity [60], shyness [61], and aggressive behavior [62]. Moreover, it has been show that IA is associated with psychiatric comorbidities among adolescence and youth population, including depression [63], anxiety disorder [64], and obsessive-compulsive personality disorder [65], but some studies also suggest that adolescents that use Internet in maladaptive way may be affected by subclinical forms of psychological problems [23]. Specifically, research has reported significant associations between both subthreshold and threshold forms of IA and emotional/psychosocial problems [31, 66], including depressive/anxiety symptoms [67], psychosomatic symptoms [68], obsessive compulsion, and interpersonal sensitivity [69]. Overall, these findings suggest that one of the primarily reasons that can lead to adolescent excessively using Internet may be to alleviate psychological suffering and difficulties [5].

On the basis of previous literature, which has underlined the correlations between the adolescents' primary relationships, their psychological difficulties, and the IA, we aim to study in a large community sample the Internet use/abuse. Indeed, to the best of our knowledge, no other studies have specifically investigated adolescents' attachment to parents and peers and its influence on adolescents' Internet use/abuse, considering their psychological profiles. Therefore, the present study aimed to investigate in a large community sample

- differences in the use/abuse of Internet, psychopathological risk, and adolescent's attachment on the basis of sex and age;
- the influence of attachment to parents and peers on use/abuse of Internet, considering the effect of adolescents' psychological profiles.

## 2. Materials and Methods

Over a period of one year, 1105 adolescents (43.6% boys and 56.4% girls) from 12 to 20 years (average age = 15.55; SD = 1.68) were recruited for this study, through the collaboration of high schools of center-south of Italy. Sample was divided into three age groups on the basis of scientific literature [14]:* early adolescence*, from 12 to 14 years (*N* = 326; 152 boys and 174 girls);* middle adolescence,* from 15 to 17 years (*N* = 434; 164 boys and 270 girls); and* late adolescence*, from 17 to 20 years (*N* = 345; 166 boys and 179 girls).

This study was approved before its start by the Ethical Committee of the Department of Dynamic and Clinical Psychology at Sapienza University of Rome, in accordance with the Declaration of Helsinki. All adolescents and their parents signed informant consent, in which the study was illustrated in detail.

Most of the adolescents recruited for the study lived in families (98.6%). 83.8% of parents are married or cohabiting (*N* = 926), while 14.8% (*N* = 163) are separated/divorced.

The adolescents who filled out the anamnestic questionnaire (purpose built for the study to evaluate the demographic data of the adolescent and his family) and accepted to participate in the study were administered the following self-reporting instruments. The order of administration was randomly decided.

### 2.1. Tools

The* Symptom Checklist-90-Revised* (SCL-90-R; [70]) is a multidimensional self-report questionnaire, composed of 90 items. It identifies the presence of psychological symptoms with a broad spectrum that may have been experienced by the subject in the last week. Each item describes a physical or psychological symptom that is evaluated on a Likert 5-point scale (0: not at all, 1: a little, 2: moderately, 3: very, 4: very much). The scores could be interpreted on nine symptomatological dimensions: somatization (SOM; indicating disorders that arise from the perception of bodily dysfunction including cardiovascular, gastrointestinal, and respiratory symptoms); obsessive-compulsivity (O-C; thoughts, impulses, and actions subjectively experienced as persistent and ego-dystonic); interpersonal sensitivity (INT; feelings of inadequacy and inferiority with respect to other people); depression (DEP; includes a broad spectrum of symptoms associated with a depressive syndrome); anxiety (ANX; general signs of anxiety such as nervousness, tension, and tremors as well as panic attacks and feelings of terror, apprehension, and fear); hostility (HOS; thoughts, feelings, and actions typical of a state of anger, irritability, and resentment); phobic anxiety (PHOB; persistent fearful response—for a specific person, specific place, object, or situation—which is recognized as irrational and disproportionate to the stimulus); paranoid ideation (PAR; corresponding to a thought disorder characterized by suspicion, fear of loss of autonomy, and hostility); psychoticism (PSY; including items indicative of an introverted, isolated lifestyle, as well as symptoms of schizophrenia, such as hallucinations and disorders of thought control). Furthermore, it is possible to score the* Global Severity Index* (GSI), indicating the total subjective distress.

Prunas et al. [71] demonstrated satisfactory internal consistency of the Italian version of the SCL-90-R in adolescents and adults (*α* coefficient, 0.70–0.96) with a clinical cut-off score ≥ 1 in GSI indicating psychopathological risk [71].

The* Shorter PROMIS Questionnaire* (SPQ; [72] Italian adaptation edited by Baiocco et al., [73]) is considered an accurate indicator of the trend towards dependent behavior. The Shorter PROMIS Questionnaire (SPQ) is a 16-scale self-report instrument assessing the use of nicotine, recreational drugs, prescription drugs, gambling, sex, caffeine, food bingeing, food starving, exercise, shopping, work, dominant and submissive relationships, and dominant and submissive compulsive helping. It consists of 160 items (10 for each scale) and a 6-point Likert scale (from 0, strongly disagreeing, to 5, strongly in agreement). The Italian version of the SPQ [73] has been adapted specifically for adolescents and evaluates also Internet, mobile, and video-games addictions. In addition, the SPQ demonstrated adequate estimates of Internal reliability. Baiocco and colleagues' results [73] suggest a good validity and reliability of the test. In the research presented we have used the specific section on Internet abuse. This subscale consists of 10 items that can be evaluated through a 6-point Likert scale (from 1, extremely false for me, to 6, extremely true for me).

The* Inventory of Parent and Peer Attachment* (IPPA; [74]) is a self- report scale that measures adolescents' perceptions of their attachment to their parents and peers. It considers feelings of security and positive/negative aspects of the affective and cognitive dimensions, presented in the relationship with parents and friends. It is composed of three parts: the first is specific for the mother, the second for the father, the third for friends. The parent parts consist of 28 items for each parent, while the section relative to friends is composed of 25 items. All three are measured on a Likert 5-point scale (from 1, never true, to 5, always true). The items measure both a global score of security attachment and three dimensions of the attachment relationship:

- alienation, which refers to the degree of anger and isolation in attachment relationships;
- communication, which refers to the quality of communication between the adolescent and attachment figures;
- trust, which refers to the degree of mutual trust between the adolescent and attachment figures in relation to the experience of feeling accepted.

Studies conducted by the authors show that adolescents who report safety concerns about parents and peers have even less conflict with parents, more satisfaction in life, more self-esteem [74], and poor stress and symptomatology (depression, anxiety, resentment, alienation, hidden anger, and loneliness) [75].

The Italian validation [76] showed a good internal consistency, ranging from .85 to .90 for trust, from .83 to .89 for communication, and from .62 to .71 for alienation.

## 3. Statistical Analysis

A preliminary screening of the data showed few data missing for each instrument (2% for each instrument). Missing data were corrected using multiple imputation in SPSS software (version 24.0).

To examine the adolescents' use/abuse of Internet, their attachment with parents and peers, and their psychological profiles, we carried out multivariate analyses of variances (MANOVAs), considering the effects of sex and age group. We considered as dependent variables the adolescents' SPQ total score, the IPPA subscales and total scores, the SCL-90-R subscales and total score, while we considered sex and age group as the independent variables. In all MANOVAs, univariate analyses were then conducted on significant effects, and the Bonferroni's test was used for contrasts.

After verifying the presence of correlations between variables, hierarchical regression analyses were conducted to verify the influence of parental and peer attachment on Internet use/abuse, considering the moderating effect of adolescents' psychopathological risk. Specifically, the IPPA instrument's subscales were used as independent variables, adolescents' SCL-90-R Global Severity Index as moderator variable, and the SPQ total score as a dependent variable. Moderation analysis was conducted utilizing the PROCESS macro for SPSS [77]. All analyses were performed with Statistical Package for the Social Sciences, SPSS software 24.0.

## 4. Results

### 4.1. Assessment of Use/Abuse of Internet, considering Adolescents' Sex and Age Group

We carried out univariate analysis of variance (ANOVA) to verify differences in Internet use/abuse in adolescence, on the basis of sex and age group. Results show that girls had higher scores than boys (*F* = 6.64; *p* < .05). Furthermore, early adolescents showed higher scores than late adolescents (*F* = 5.31; *p* < .01). No significant differences in scores were found on the basis of sex *∗* age groups (*F* = 4.43; *p* > .05).  Table 1 shows mean and standard deviation of adolescents' use/abuse on Internet, on the basis of sex and age group.

### 4.2. Assessment of Adolescents' Attachment to Parents and Peers, considering Adolescents' Sex and Age Group

We carried out multivariate analysis of variance (MANOVA) to verify differences in adolescents' attachment to parents and peers on the basis of sex and age groups.

Results show the presence of significant differences on the basis of sex (Lambda = .85; *F* = 21.24; *p* < .001). Univariate analyses were conducted on significant effects, and the Bonferroni's test was used for contrasts. Mean and standard deviations of adolescents' attachment to mother, father, and peers on the basis of sex and age group are illustrated in Table 2.

Results of univariate analyses show that girls had higher scores than boys on the scales father alienation (*F* = 15.71; *p* < .001), mother alienation (*F* = 11.37; *p* < .01), mother communication (*F* = 13; *p* < .001), peers communication (*F* = 83.29; *p* < .001), and peers trust (*F* = 25.18; *p* < .001).

### 4.3. Assessment of Adolescents' Psychological Profiles, considering Adolescents' Sex and Age Group

We carried out multivariate analysis of variance (MANOVA) to verify differences in adolescents' psychological profiles on the basis of sex and age groups.

Results show the presence of significant differences on the basis of sex (Lambda = .85; *F* = 17.28; *p* < .001) and age group (Lambda = .95; *F* = 2.6; *p* < .001).

**Figure 1:**
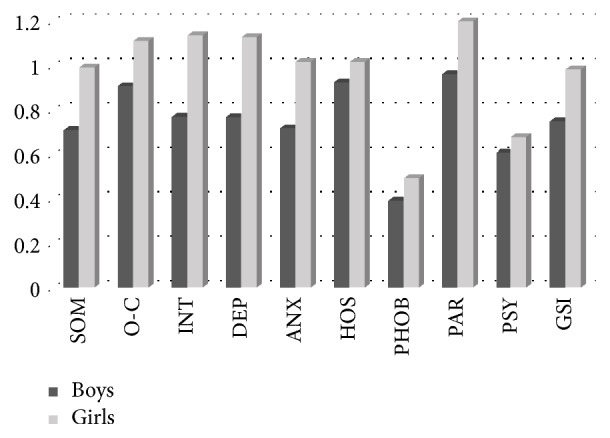
Differences of mean scores of SCL-90-R subscales between boys and girls.

Univariate analyses were conducted on significant effects, and the Bonferroni's test was used for contrasts. Results of univariate analyses show that girls had higher scores than boys on all SCL-90-R subscales (see Figure 1).

Regarding the effect of age groups, results show differences in SCL-90-R subscales: obsession-compulsivity (*F* = 6.31; *p* < .01), depression (*F* = 5.95; *p* < .01), hostility (*F* = 5.23; *p* < .01), paranoid ideation (*F* = 7.07; *p* < .01), and Global Severity Index (*F* = 3.71; *p* < .05). Bonferroni post hoc test showed that late adolescents had higher scores than early adolescents on SCL-90-R subscales: obsession-compulsivity (*p* < .01), depression (*p* < .01), paranoid ideation (*p* < .01), and Global Severity Index (*p* < .05). Furthermore, late adolescents showed higher scores than other groups on the subscale hostility (*p* < .05).  Table 3 shows mean and standard deviations of adolescents' psychological profiles, on the basis of sex and age group.

### 4.4. Assessment of the Influence of Attachment to Parents and Peers on Internet Use/Abuse

In order to evaluate the influence of attachment on Internet use/abuse, considering the effect of psychological profiles, hierarchical regression analyses were conducted. Specifically, at Step 1 the SCL-90-R GSI and the IPPA subscale scores for fathers, mothers, and peers have been included as independent variables and the SPQ total score as a dependent variable. At Step 2, the interaction between adolescents' GSI and IPPA subscales was inserted in the model as independent variable, to verify moderator effect. Standardized scores were used.

At Step 1, analysis shows that Internet use/abuse was predicted by adolescents' attachment to mothers (Beta = −.182; *t* = −6.220; *p* < .001) and fathers (Beta = −.163; *t* = −5.560; *p* < .001) but not by adolescents' attachment to peers (*p* > .05).

At Step 2, results show that adolescents' Global Severity Index moderated the influence of attachment to mother on Internet use/abuse (*R*^2^ = .157; *F* = 69.66; *p* < .001), but this moderating effect was not found in the influence of attachment to father and peer on Internet use/abuse (*p* > .05).

Consequently, we use the PROCESS macro for SPSS [77] to verify moderating effects. Main and interaction effects were centered to minimize multicollinearity [78].

Results show an attachment to mother × SCL-90-R Global Severity Index interaction effect in predicting use/abuse of Internet (*F*Δ(3,1101) = 69.66, *R*^2^Δ = .16, *p* < .001). Conditioned at 1 standard deviation below the mean on Global Severity Index, attachment was inversely related to use/abuse of Internet (*t* = −6.16, *p* < .001), and when conditioned at 1 standard deviation above the mean on GSI, attachment was inversely related to use/abuse of Internet (*t* = −3.26, *p* < .05). In fact, test of highest order unconditional interaction showed *R*^2^chng = .0042; *F* = 5.53; *p* = .019.

Thus, a high attachment to the mother predicted less Internet use/abuse when adolescents presented a low psychopathological risk. However, the effect of attachment to the mother on less Internet use is still present even in the case of adolescents' higher psychopathological risk, even if this effect is reduced.

## 5. Discussion

The present paper aimed to assess Internet use/abuse in adolescents from a community sample. In particular, our study aimed to investigate differences between boys and girls in different development stages in Internet use/abuse, their attachment to parents and peers, and their psychological profiles. Furthermore, we wanted to verify whether attachment to parents and peers influenced adolescents' Internet use/abuse, considering adolescents' psychopathological risk.

Regarding the first objective, we carried out multivariate analysis of variance (MANOVA) to verify differences between boys and girls in different development stages in Internet use/abuse. Results showed that girls had higher levels of Internet use/abuse than boys. International scientific literature in this field is contrasting. Although previous researches have evidenced a higher risk of Internet overuse among boys [79, 80], other studies have reported no gender differences in problematic Internet use [81]. These conflicting results may depend on the different research objects (e.g., use of video games, social networks). In our study, we have evaluated Internet use, in its various functions and, as suggested by Karacic and Oreskovic [82], the percentage of female adolescents being Internet addicted is increasing.

Furthermore, analysis showed that early adolescents show higher scores than late adolescents on Internet use/abuse. Previous research has reported mixed findings: some studies have underlined a significant higher incidence of Internet use/abuse during middle adolescence [83] or late adolescence [52], but other studies did not report age differences [84, 85]. In our sample, early adolescents reported higher levels of use/abuse of Internet. This result may depend on changes occurring during early adolescence and the immaturity of self-regulation abilities, factors that may increase vulnerability to addictions [86, 87].

Moreover, analysis showed that girls had higher levels of communication with mothers than boys, but also higher levels of alienation with fathers and mothers. This is consistent with previous studies that have reported a perception of higher quality of communication with mothers [88] and higher levels of alienation to parents [89] among female adolescents, suggesting that girls are more susceptible to parental response, especially maternal.

Regarding peers, girls showed more trust and communication. This is in line with previous studies that have reported a higher level of peer attachment among teenage girls [90, 91]. Some authors have explained these gender differences on the basis of evidence that girls are more relationship oriented, and they search for closer peer relationships to share emotional issues. In contrast, boys are more object oriented and form relationship primarily to share activities [92].

Interesting data have emerged as regards psychopathological profiles. In fact, analysis showed that girls had more maladaptive psychological profiles than boys on all areas. Recent studies have evidenced that girls reported higher psychopathological symptoms than boys, especially in internalizing area [93], but our results expand these findings also for externalizing problems. This is in accordance with Ara's study [94] who reported a cooccurrence of internalizing and externalizing problems especially among female adolescents.

As regards developmental stage, results showed that late adolescents had higher levels of depression, obsession-compulsivity, paranoid ideation, and hostility than early adolescents and confirm previous studies that have underlined an increase in psychopathological difficulties during the development [95].

International studies have widely evidenced that during adolescence emotional-behavioral functioning is hyperactivated [96] and many young people may excessively use Internet to cope with negative emotions resulting from attachment situations and to lighten psychological suffering [42, 97]. Indeed, many studies have shown that a low quality of parental attachment is related to adolescent behavior addiction, including IA [42, 44]. Also, several empirical studies have evidenced that quality of attachment to parents and peers was associated with adolescent's psychological adjustment [98, 99] and that these factors influence each other.

On the basis of these theoretical and empirical premises and in order to be able to assess the possible influence of attachment to parents and peers on the use and abuse of Internet, hierarchical regression analyses were conducted, assessing the moderating effect of the psychopathological risk.

Analysis showed that adolescents' attachment to parents (but not to peers) influenced Internet use/abuse. Furthermore, moderation analysis suggested that adolescents' psychopathological risk had a moderating effect on the relationship between attachment to mothers and the use of Internet. Although this result is significant, the effect size is not great (*R*^2^chng = .0042). In fact, results showed that a higher attachment of adolescents to the mother predicted less Internet use/abuse, but this predictive effect is reduced by the presence of adolescents' psychopathological risk.

Overall, our results evidenced the crucial role played by attachment on adolescent's Internet use. These findings suggest that unpleasant feelings of isolation, anger, or detachment experienced in attachment relationships with parents may predispose adolescents to cope with these emotions through an excessive use of Internet, in order to avoid and/or reduce the distress resulting from adverse attachment experiences [43, 45].

This study has some limitations. In fact, some individual variables of the adolescent were not investigated, such as traumatic events that may have been experienced by young people [100, 101] and the levels of impulsivity [60]. Furthermore, we assessed adolescents' attachment to parents, but we did not evaluate mothers' and fathers' psychopathological risk [102–105]. Notwithstanding the above limitations, the present study has several strengths. In particular, the sample size allows having numerous data on this specific age group. Furthermore, attachment has been investigated both in primary relationships with parents and in relations with peers, allowing a broad view of the relational qualities of adolescents.

In light of the results, we believe that further studies are important to investigate more individual variables of teenagers, in order to allow greater understanding, so that appropriate prevention and treatment programs may be implemented.

## Figures and Tables

**Table 1:** Mean and standard deviations of adolescents' scores of Internet use/abuse, on the basis of sex and age group.

	Early adolescence		Middle adolescence		Late adolescence	
	Boys	Girls	Boys	Girls	Boys	Girls
Internet use/abuse	24.64 (9.24)	27.4 (9.5)	23.04 (8.32)	25.49 (9.11)	24.39 (9.46)	23.45 (8.17)

**Table 2:** Mean and standard deviations of adolescents' attachment to mother, father, and peers, on the basis of sex and age group.

	First adolescence		Middle adolescence		Late adolescence	
	Boys	Girls	Boys	Girls	Boys	Girls
Father alienation	18.66 (6.49)	20.31 (6.83)	18.73 (6.91)	19.83 (6.81)	18.37 (6.09)	20.47 (6.54)
Father communication	32.36 (8.17)	30.5 (8.75)	31.49 (8.5)	31.6 (9.52)	32.72 (7.38)	31.55 (8.67)
Father trust	37.75 (8.28)	37.77 (8.58)	38.29 (8.19)	38.18 (8.44)	38.61 (7.39)	37.98 (8.25)
Mother alienation	17.78 (5.9)	20.01 (6.82)	18.24 (5.85)	19.01 (6.33)	18.6 (6.27)	19.46 (6.14)
Mother communication	33.99 (7.77)	34.83 (8.22)	33.49 (7.04)	35.98 (8.39)	33.58 (7.54)	35.63 (8.79)
Mother trust	40.33 (7.04)	39.14 (7.64)	39.78 (6.68)	40.1 (7.41)	39.49 (7.1)	39.77 (7.27)
Peers alienation	17.45 (4.88)	16.29 (4.31)	16.4 (4.96)	15.81 (4.41)	16.03 (4.59)	17.04 (4.26)
Peers communication	26.94 (6.71)	30.69 (6.13)	28.01 (6.86)	32.39 (4.99)	28.98 (6.05)	31.07 (6.13)
Peers trust	39.03 (7.63)	41.28 (7.3)	39.48 (8.01)	42.91 (5.86)	40.02 (7.53)	41.04 (7.48)

**Table 3:** Mean and standard deviations of adolescents' scores at SCL-90-R subscales, on the basis of sex and age group.

	First adolescence		Middle adolescence		Late adolescence	
	Boys	Girls	Boys	Girls	Boys	Girls
SOM	.66 (.62)	.96 (.73)	.71 (.66)	.97 (.72)	.75 (.62)	1.04 (.73)
O-C	.75 (.63)	1.08 (.76)	.91 (.75)	1.07 (.72)	1.04 (.72)	1.19 (.71)
INT	.65 (.64)	1.18 (.85)	.76 (.71)	1.11 (.76)	.89 (.76)	1.13 (.74)
DEP	.63 (.6)	1.07 (.75)	.79 (.73)	1.11 (.72)	.87 (.67)	1.21 (.71)
ANX	.63 (.6)	.98 (.72)	.73 (.68)	.99 (.67)	.78 (.66)	1.1 (.75)
HOS	.76 (.75)	1.01 (.86)	.93 (.88)	.95 (.78)	1.06 (.89)	1.12 (.89)
PHOB	.4 (.59)	.56 (.67)	.39 (.59)	.48 (.52)	.38 (.54)	.45 (.55)
PAR	.76 (.74)	1.18 (.79)	.96 (.9)	1.16 (.8)	1.15 (.89)	1.27 (.82)
PSY	.53 (.62)	.72 (.65)	.6 (.63)	.64 (.61)	.68 (.62)	.7 (.62)
GSI	.65 (.55)	.98 (.63)	.75 (.62)	.95 (.56)	.84 (.57)	1.03 (.58)
